# Propensity Score and Instrumental Variable Techniques in Observational Transplantation Studies: An Overview and Worked Example Relating to Pre-Transplant Cardiac Screening

**DOI:** 10.3389/ti.2022.10105

**Published:** 2022-06-27

**Authors:** Ailish Nimmo, Nicholas Latimer, Gabriel C. Oniscu, Rommel Ravanan, Dominic M. Taylor, James Fotheringham

**Affiliations:** ^1^ Renal Department, Southmead Hospital, North Bristol National Health Service Trust, Bristol, United Kingdom; ^2^ School of Health and Related Research, University of Sheffield, Sheffield, United Kingdom; ^3^ Transplant Unit, Royal Infirmary of Edinburgh, Edinburgh, United Kingdom

**Keywords:** observational studies, causal inference, confounding, propensity score, instrumental variable

## Abstract

Inferring causality from observational studies is difficult due to inherent differences in patient characteristics between treated and untreated groups. The randomised controlled trial is the gold standard study design as the random allocation of individuals to treatment and control arms should result in an equal distribution of known and unknown prognostic factors at baseline. However, it is not always ethically or practically possible to perform such a study in the field of transplantation. Propensity score and instrumental variable techniques have theoretical advantages over conventional multivariable regression methods and are increasingly being used within observational studies to reduce the risk of confounding bias. An understanding of these techniques is required to critically appraise the literature. We provide an overview of propensity score and instrumental variable techniques for transplant clinicians, describing their principles, assumptions, strengths, and weaknesses. We discuss the different patient populations included in analyses and how to interpret results. We illustrate these points using data from the Access to Transplant and Transplant Outcome Measures study examining the association between pre-transplant cardiac screening in kidney transplant recipients and post-transplant cardiac events.

## Introduction

Randomised controlled trials (RCTs) are the gold standard study design for determining causal associations between clinical interventions and outcomes (1, 2). In transplantation, RCTs have shaped immunosuppression practice (3, 4), informed the management of cardiovascular risk (5), and guided infection prophylaxis (6). By randomly assigning individuals to treatment or control groups, two populations with similar characteristics are created, meaning differences in outcome likely result from differences in treatment.

In some situations RCTs are inappropriate or impractical, for example if there are ethical concerns or excessive costs (7). In transplantation, the small numbers of recipients compared to general populations can make achieving required sample sizes for small treatment effects challenging. Further, standard practice (often used as the comparator in RCTs) varies between centres, the time between waitlisting and transplantation may necessitate long follow up, and the lack of control over transplant timing can put pressure on the informed consent process (8). If individuals recruited to trials are healthier or sicker than the overall population, results may also not be generalisable.

When RCTs are impractical, observational data can inform practice. However, as the exposure is not randomly assigned, differences in case-mix can occur between exposed and unexposed groups. This generates confounding bias: a situation where the treatment and outcome have a common cause, resulting in a lack of exchangeability between treated and untreated groups. This can result in the association between treatment and outcome differing from the true effect measure (9). Confounders are identified using causal diagrams that depict potential pathways between treatment and outcome (10, 11). However, only known confounders can be adjusted for in multivariable regression models and unmeasured confounding can persist. Further, multivariable models may be overfitted if the number of covariates is large relative to the number of outcome events. To minimise confounding and improve the validity of causal inference from observational studies, propensity score and instrumental variable analyses are increasingly being used (12). These techniques do not minimise other forms of bias that make emulating an RCT from observational data challenging (13, 14), so whilst they have advantages over traditional methods they don’t solve all issues with observational studies.

In kidney transplantation, there is no contemporary RCT examining the utility of screening for asymptomatic coronary artery disease prior to transplant listing. Screening is frequently performed but there is variation in practice between centres, likely influenced by local opinion (15). An RCT to examine if screening before transplant listing reduces post-transplant cardiac events would be challenging (16). Individuals would need to be identified at the point of screening, far in advance of transplantation. The low cardiac event rate would necessitate a large study population and high recruitment rates (17) which may be difficult to achieve if there is anxiety around recruiting patients, especially higher-risk individuals, meaning a study may be underpowered or not have generalisable results.

Given these challenges, we use observational data from the Access to Transplant and Transplant Outcome Measures (ATTOM) study (18) on pre-transplant coronary artery disease screening to describe the principles and assumptions of propensity score matching, inverse probability weighting, and instrumental variable analyses. We illustrate how these techniques are performed and interpreted and compare their results.

## The Propensity Score

The propensity score (PS) refers to the predicted probability of an individual receiving a treatment by collapsing measured confounders into a single value, ranging from 0: no probability to 1: absolute probability of them receiving the treatment of interest (19).

The PS is typically estimated using a logistic regression model specifying the exposure as the dependent variable and measured confounders as independent variables. Measured confounders are those known at baseline that are predictive of both treatment and outcome. Variables that are predictive of treatment but not outcome should not be included as this may increase the variance of the estimated exposure effect (20). Confounders should not be chosen based on a statistically significant association with the exposure but based on prior knowledge and clinical judgement as formalised and summarised in a directed acyclic graph (10, 11, 20).

Once the model has been created, each individual’s PS is generated based on their measured confounders. The score reflects their propensity for receiving the treatment, not whether this actually happened. Two balanced groups with a similar distribution of PS can then be created using matching or weighting techniques. Key features of PS analyses are shown in Table 1, and a detailed description of PS assumptions is in Supplementary Table S1.

**TABLE 1:** Comparison of propensity score and instrumental variable techniques.

	Propensity score matching	Propensity score weighting	Instrumental variable
Assumptions	Positivity	Positivity	Relevance assumption
	Exchangeability/ignorability	Exchangeability/ignorability	Exclusion restriction
	Consistency	Consistency	Independence assumption
			Monotonicity or homogeneity
Unmeasured confounding	Not eliminated	Not eliminated	Eliminated/reduced
Study application	Smaller studies or low event rate	Smaller studies or low event rate	Large multi-centre studies
Analysis and interpretation	Patient-level	Patient-level	Instrument level e.g. centre, physician
Causal effect	Average treatment effect on the treated	Average treatment effect	Average treatment effect or local average treatment effect depending on assumptions
Advantages	Simple to analyse and interpret	Retains data from all patients	Does not require modelling on confounders, minimises unmeasured confounding
Disadvantages	Exclusion of unmatched patients means results may not be applicable to whole study population	Results can be unstable if extreme weights are present	Analysis assumptions difficult to test Challenging to find suitable instrument

### Propensity Score Matching

**FIGURE 1:**
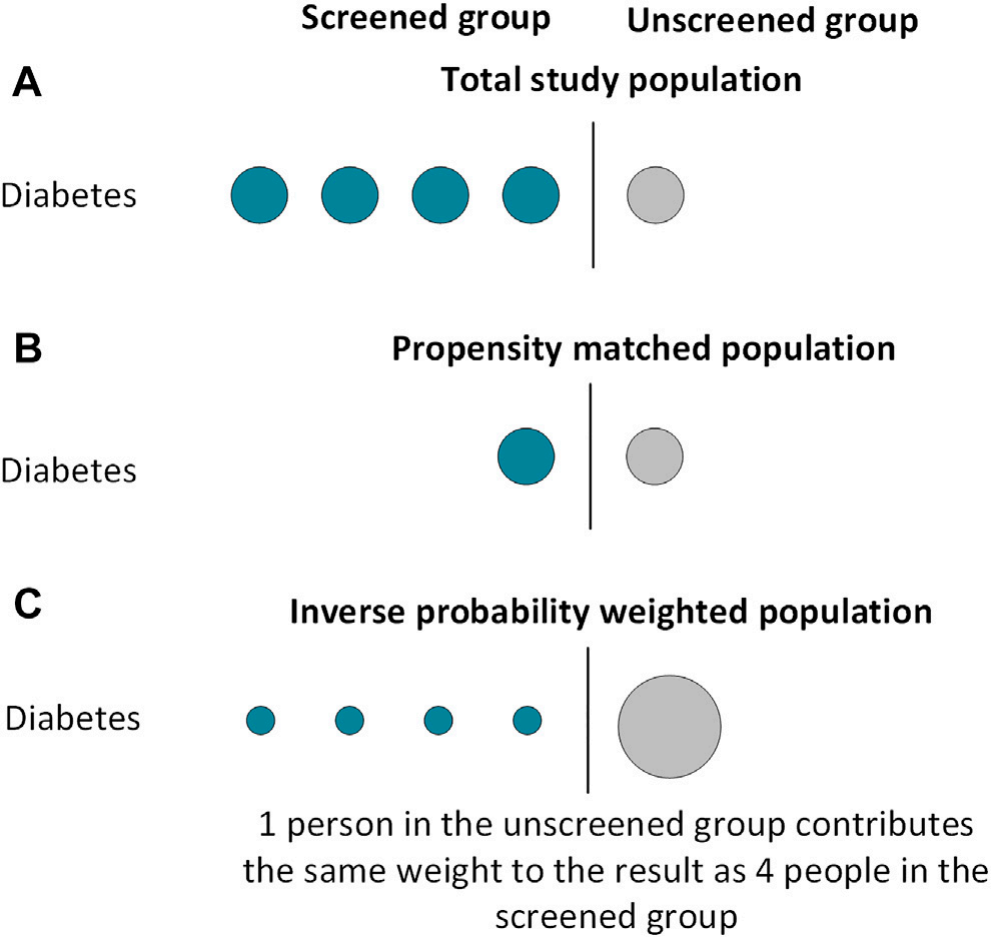
Included subjects in propensity score analyses using matching and weighting techniques.

In propensity score matching, treated and untreated individuals are “paired” based on their PS (Figure 1). Depending on the prevalence of the treatment, individuals can be matched on a 1:1 or 1:many basis. Nearest-neighbour matching identifies pairs with the closest PS. In “matching without replacement,” an individual can only be matched once before being removed from the matching pool. This means pairs generated later in the matching process may have larger differences in their PS (21). Matching with replacement allows control patients to be matched to more than one treated patient. An alternative to nearest-neighbour matching is optimal matching, which minimises the difference in PS between pairs across the whole population. In large populations, nearest-neighbour and optimal matching give similar results (22). Both techniques include a “caliper” to avoid the inclusion of poorly matched pairs. This specifies the maximum acceptable difference in PS for a pair to match, generally accepted as 0.2 times the standard deviation of the logit of the PS to provide the optimal balance of matching quantity and quality (23, 24). Individuals who are unmatched are excluded from further analyses. In practice, as it isn’t always clear what the “ideal” statistical method is, performing analyses using a number of these techniques can help assess how sensitive results are to method specification.

The matching technique should create two groups with an equal distribution of measured covariates (Figure 1). The balance of covariates between groups can be examined using standardised differences, calculated by dividing the difference in proportion (for binary variables) or sample mean (for continuous variables) by the pooled standard deviation. There is no definite consensus on an acceptable standardised difference; a value below 0.1–0.2 is generally accepted (25). Visual diagnostic tools can also be used to examine covariate balance, as demonstrated in our worked example (26). Once the groups are balanced, they can be compared using standard regression analyses. These analyses can be univariable or multivariable, with the multivariable technique including the variables used to generate the PS. A multivariable model compensates for imperfect covariate balance and, if specified correctly, minimises the risk of a biased estimator (27). However multivariable models lose the advantage of having only 1 covariate in the final model, so could be overfitted if the number of covariates is large relative to the number of outcome events. Further, in the event of misspecification of the PS model, this method could increase bias (28).

### Inverse Probability Weighting Using Propensity Scores

Inverse probability weighting (IPW, also known as propensity score weighting) creates a pseudo-population informed by all patients with a balanced distribution of measured covariates between groups (29). By doing so, IPW avoids excluding individuals from analyses and may result in better covariate balance than PS matching (30).

Each individual is assigned a “weight” depending on their measured covariates and the treatment they receive. For individuals who receive treatment, their weight is 1/PS, whilst individuals who do not receive treatment have a weight of 1/(1-PS). This means individuals receiving an “unexpected” treatment contribute larger weights to the analysis than individuals receiving their “expected” treatment (Figure 1). Each crude weight is greater than or equal to 1. If some patients have large weights, this can make results unstable. To minimise this risk, weights are frequently “stabilised” before further analysis. This is relevant if a multivariable regression model is being used; stabilisation does not affect univariable models which contain only the treatment indicator (31). Stabilisation involves multiplying the weight by the proportion of exposed patients for the treated group, and by the proportion of unexposed patients in the untreated group (32). Once stabilised, the mean weight for the population should be approximately equal to 1. A regression analysis where each individual is weighted by their inverse probability of receiving treatment can then be performed. As with PS matched analyses, this regression can be univariable or multivariable. The same caveats of the multivariable model in PS matched methodology apply to IPW analyses.

### Strengths of Propensity Score Analyses

PS techniques have several advantages over conventional multivariable regression models. First, conventional multivariable Cox models require around 10 events per covariate to produce a stable estimate, and combining covariates into a single PS is useful when the population is small, event rate is low, or number of covariates is large (33, 34, 35).

Second, in conventional regression models the treated and untreated groups can systematically differ. This means estimating the effect of treatment on a patient, who would never have been considered for treatment in real life, can be unreliable as the estimation is based on model extrapolations beyond the support of the data. PS matched analyses refer to only those patients who could feasibly exist in either the “treated” or “untreated” group. Whilst PS matched analyses can therefore provide improved real-world results, identifying the population to whom the results are applicable to can be challenging, especially where there is variation in treatment practice between centres.

Third, PS models highlight the limitations within which results should be interpreted. If a large proportion of individuals are unmatched in PS matched analyses, or there are patients with large PS weights in IPW analyses, this signifies poor overlap in covariate distributions between treated and untreated groups and means the likelihood of individuals being allocated to either treatment group is low. As traditional multivariable models extrapolate results to individuals in under-represented covariate strata, this could lead to bias in effect estimates. PS methods can alert researchers to these issues and highlight the limits within which comparisons of treatment options can be made.

### Limitations of Propensity Score Analyses

PS assumptions (exchangeability, positivity, and consistency) are described in Supplementary Table S1, and it may be difficult to prove these assumptions hold. If the treatment is rare, there may be insufficient data to generate the PS. Further, the PS only encompasses measured confounders. Confounders that are unknown, poorly recorded, or not measurable cannot be controlled for and may not be balanced between groups, leading to unmeasured confounding bias.

In PS matching, unmatched individuals are “lost,” reducing the study size. Individuals with the highest and lowest PS (the “always treated” and “never treated”) are less likely to be matched and are under-represented in the regression models. Whilst there is no “required” proportion of patients that must be matched, the causal effect is only applicable to matched patients, not the whole study population.

In IPW, data from all participants is retained. However, if individuals contribute large weights to analyses, results may be unstable. There is no consensus on what a “large” weight is, and weight stabilisation is often used to minimise this risk. Some advocate truncating weights to a maximum of 10 for more precise estimates, (36) but this may re-introduce some of the confounding that the method aims to remove.

For interested readers, more detailed information on propensity scores can be found at the following references (9, 37, 38, 39).

## Instrumental Variable Analysis

Instrumental variable (IV) analyses were developed for economic studies and subsequently adopted in the medical setting. They aim to minimise confounding by indication by examining individuals based on an “instrumental variable”: a variable that influences treatment and has no confounder with the outcome. This allows the IV to be capitalised on as a type of natural randomisation (40). Individuals are analysed according to the instrument rather than by the treatment they receive akin to an intention to treat analysis, whereby individuals in RCTs are analysed according to their randomisation group rather than by received treatment. Their advantage is they do not assume an absence of unmeasured confounders to the treatment-outcome relationship, allowing an independent treatment effect to be estimated as in an RCT. Key features are shown in Table 1.

**FIGURE 2:**
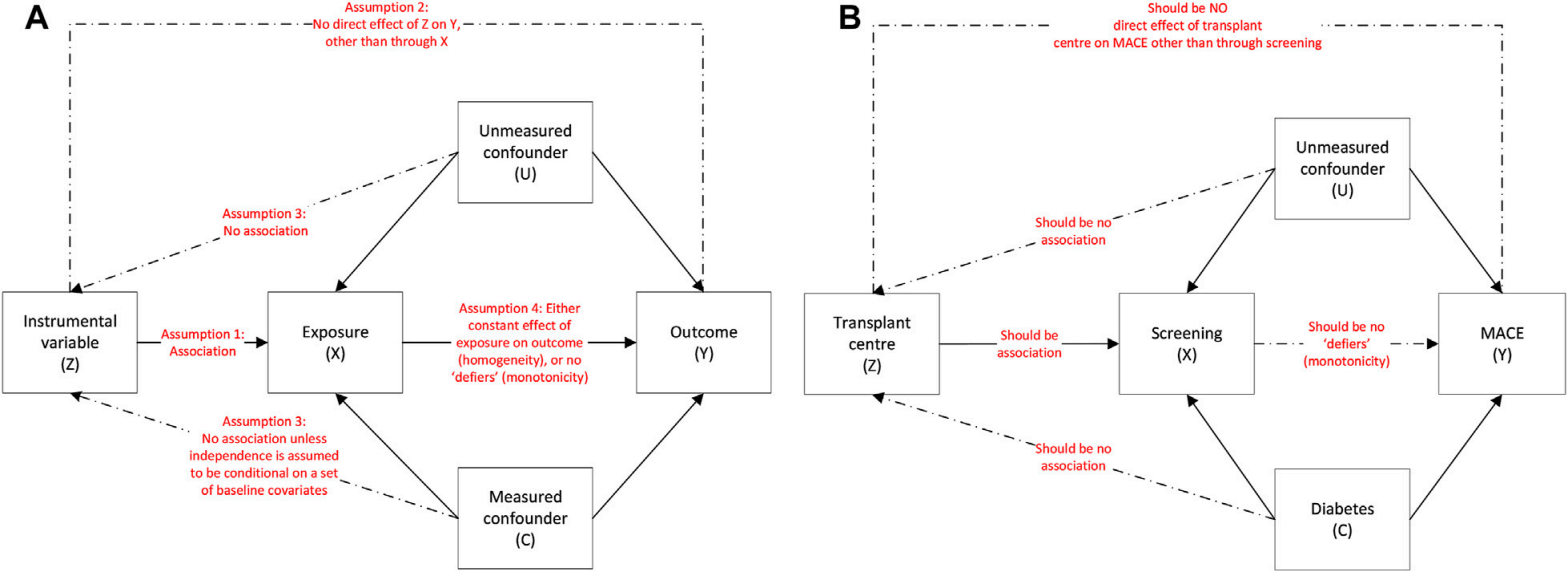
**(A)**: Instrumental variable assumptions and the associations between the instrumental variable (Z), exposure (X), outcome (Y), measured confounders (C) and unmeasured confounders (U) and **(B)**: using the example of screening on MACE.

To perform IV analyses, the IV is recommended to meet key assumptions (Figure 2A): (41).

(1) It must be strongly associated with the exposure (relevance assumption).
(2) It must only affect outcome through its association with the exposure (exclusion restriction).
(3) There must be no unmeasured confounders to the instrumental variable and the outcome (independence assumption).
(4) A fourth assumption is either that of effect homogeneity or effect monotonicity. Effect homogeneity states that the treatment should have a constant effect on the outcome across all individuals. In effect monotonicity, no patients should receive the opposite treatment to expected at all levels of the instrument i.e., at both the instrument to which they were assigned and instrument(s) to which they were not assigned (so called “defier” patients; Supplementary Figure S1) (9, 42). Identifying which “compliance type” a patient belongs to however is impossible. Further, when instruments are multi-categorical or preference-based, even defining compliance types (and thus effect monotonicity) is complex and can limit the clinical applicability of results.

A potential IV is initially identified using empirical evidence. The analysis then involves a two-stage regression model. As the technique originated in economics this was traditionally two sequential linear regressions using a two-stage least squares procedure (41). In medical studies the outcome cannot always be assessed using linear regression so here we simply refer to the technique as a two-stage instrumental regression method. In the first stage, the exposure (treatment) is regarded as the outcome and predicted from a regression model containing the instrument as an independent variable alongside other covariables. A linear regression is frequently used for the first stage even if the exposure is binary, though if the model contains additional covariates the predicted treatment value can lie outwith the range 0–1 (43). As such a linear model is only advised if few additional categorical covariates are added to the model (44).

In the second stage, a regression model examines the outcome of interest as the dependent variable, and the “predicted treatment” generated in the first stage is included as an independent variable instead of the received treatment (“predictor substitution” method). This regression can be univariable or multivariable. A multivariable model enables adjustment for potential confounding of the instrument-outcome relationship. Whilst instrument-outcome confounding represents a violation of the independence assumption, conditioning on pre-exposure covariates in the first and second stages of the IV model can reduce the impact of this and also increase the plausibility of the homogeneity assumption. (9) As such, multivariable models which include confounders of the instrument-outcome (in addition to treatment-outcome) relationship may be beneficial. Other methods of estimating the predicted treatment variable, how to include it in the second stage model, and type of second stage model exist. Broadly speaking, population effects can be interpreted using a range of first-stage regression techniques and a second-stage Cox model with the predictor substitution approach is a straight forward method for time-to-event analyses, though Cox models are not universally recommended in IV analyses unless the outcome is rare due to their potential to introduce bias (45-51).

As the analysis is performed, potential violations of IV assumptions should be assessed. Results must be interpreted in the context of how likely it is for the assumptions to be met.

(1) Relevance assumption: this is examined using the F statistic and partial R-squared values. An F statistic under 10 typically is used to identify a weak instrument (52). The greater the partial R-squared the greater the contribution of the instrument to treatment allocation, however this value varies with sample size and there is no consensus on what a satisfactory value is (53).
(2) Exclusion restriction: there is no statistical test to definitively confirm that the IV does not influence the outcome other than through treatment allocation. (54). Examining the association between the IV and the outcome can provide information on how likely a direct association is but requires careful conduct and interpretation.
(3) Independence assumption. This cannot be tested and is usually argued based on empirical evidence.
(4) Effect monotonicity or homogeneity. These assumptions may be implausible and are complex to define and assess. In effect monotonicity, identifying which compliance group (Supplementary Figure S1) a patient belongs to is impossible, and even defining compliance groups is challenging in the case of multi-categorical instruments (42).

### Limitations

Finding a suitable IV can be challenging and large multicentre studies are often required. Ensuring assumptions of the IV are met may not be possible (55). Weak instruments may also amplify bias through violation of the exclusion restriction or independence assumption and result in more biased estimates than other analyses (9). Finally, whilst IV analyses can overcome unmeasured confounding, they are less precise as individuals are examined based on estimated not actual exposure (56).

## Interpreting Results From Causal Inference Models

### Average Treatment Effects

When analysing causal inference studies, it is necessary to consider to whom the causal effect is applicable to. Terms used include the “average treatment effect” (ATE), “average treatment effect on the treated” (ATT) and “local average treatment effect” (LATE).

ATE refers to the effect of treatment on the whole population. This is typically estimated by IPW techniques, which include all study participants. ATT refers to the effect of treatment on only those individuals potentially eligible to receive it and is typically estimated by PS matched analyses. In IV analyses, the causal effect depends on whether effect homogeneity or monotonicity hold. If homogeneity is assumed, the estimate refers to the ATE. If monotonicity is assumed, the estimate refers to the LATE. This reflects the effect of treatment on the subgroup of “complier” patients who receive the expected treatment given their instrument (Supplementary Figure S1). As complier patients cannot be identified from within the study population, the LATE has limitations in informing practice/policy decisions.

As the ATE, ATT and LATE refer to different groups of patients, their effect sizes can differ. Differences can aid the interpretation of study findings by providing insights into the effect of treatment on different groups of patients, and do not necessarily signify failure of a technique.

### Conditional and Marginal Treatment Effects

In each of the above analyses, the final regression model that generates the causal effect can either be “marginal” or “conditional.” Models which contain only the treatment (or predicted treatment in the IV analysis) and outcome generate marginal treatment effects. Although the characteristics of treated and untreated individuals should be similar through the PS matching, IPW or IV techniques, generating truly “exchangeable” groups of treated and untreated patients remains difficult. Models which condition on (and hence adjust for) confounders in the final regression may reduce such residual imbalances and generate conditional treatment effects.

The effect sizes from marginal and conditional regression models differ and cannot be directly compared (57, 58). If the model has been correctly specified, marginal models estimate the average effect of treatment on the population (i.e., the effect of moving the population from being untreated to treated), whilst conditional effects are more individualised and apply to groups of patients within covariate levels (i.e., the effect of moving an individual person from being untreated to treated). Marginal treatment effects are frequently used for health policy decisions, whilst conditional treatment effects are helpful at an individual patient level. Further, even if conditional models from PS matching, IPW and IV techniques contain the same variables, unavoidable differences between analyses mean results are still not directly comparable. For example, PS matching is conditional on the covariates and the PS, whereas the other analyses are just conditional on the covariates.

## Does Screening for Coronary Artery Disease Reduce Post-Transplant Cardiac Events?

To demonstrate the above techniques, a worked example is provided using data from the ATTOM study. ATTOM was designed to examine factors associated with transplantation in the UK, recruiting patients between 2011 and 2013 (59). Data on transplant assessment was collected for patients who were waitlisted or transplanted at study recruitment. In this analysis, individuals receiving a kidney transplant between 1st November 2011 and 31st December 2017 were included. This patient selection has implications on other forms of bias in the study, outlined in Table 2.

**TABLE 2:** Design of a potential randomised control trial to investigate the utility of cardiac screening prior to kidney transplant listing, and the design of the worked example, highlighting areas of residual bias.

Component	Ideal randomised control trial	Worked example and residual bias
Eligibility	Individuals with chronic kidney disease being worked up for kidney transplantation	Patients who were recruited to the ATTOM study and received a kidney transplant. Whilst these patients are representative of the UK kidney transplant population, information was not available on all patients who commenced transplant workup and it is not known if results are applicable to this whole population. Selection bias and survivor bias may be present
Treatment strategies	Receive a cardiac screening test (and any subsequent recommended cardiac intervention) vs. not receive a cardiac screening test prior to kidney transplant listing	Receiving a cardiac screening test (and any subsequent recommended cardiac intervention) as per local standard practice vs. not receiving a screening test prior to kidney transplant listing
Treatment assignment	Eligible individuals would be randomly assigned to one of the two treatment strategies and would be aware of the treatment which they were assigned to	Patients were selected for screening based on pre-determined local protocols or clinical judgement of the medical team. As treatment assignment was not randomised and there were not strict eligibility criteria, inferences are limited to those patients who might be considered for screening, rather than patients who would never or always be screened
Follow up	Follow up would start at the time of assignment to a treatment strategy (i.e. when randomised to receive cardiac screening or not) and would continue for a set period of time over which some patients would be activated on the waitlist and receive a transplant. This is likely to require long follow up, for example 3–5 years	Follow up started at the point of kidney transplantation and was for up to 5 years. This start point was chosen as the date transplant workup commenced was unknown, and data were not available on patients who commenced workup but were not waitlisted. This risks survival bias as all patients survived until the point of transplantation. Further, the misalignment of treatment assignment and follow up start means there could be fundamental differences between patients who are transplanted after screening vs. those transplanted without screening. As screening may not have a uniform effect on individuals unobserved in this study, there is a risk of selection bias
Primary end point	Post-transplant MACE. The exact time frame post-transplant that should be examined could be debated, but given screening aims to reduce short-term morbidity and mortality a time frame of around 1 year could be considered	Post-transplant MACE at 90 days, 1 year and 5 years post-transplant. Patients were censored for non-cardiac death, therefore estimates refer to the direct effect of screening on MACE and not the total effect of screening on MACE through all causal pathways, including through any effect on non-cardiac death
Secondary end point	Activation on transplant waitlist	Not captured
	Time to waitlisting	
	Time to transplantation	
	Waitlist MACE	
	Patient reported outcomes	
Causal contrast	Intention-to-treat effect—effect of being randomised to screening or no screening, even if off-protocol screening tests were performed	Per protocol effect—effect of adhering to the treatment strategies over follow up
	Per protocol effect - effect of adhering to the treatment strategy over follow up	
Statistical analysis	Intention-to-treat; consideration would need to be made as to how to analyse patients not transplanted over follow up	Per protocol analysis

We wished to examine whether cardiac screening reduced post-transplant major adverse cardiac events (MACE). MACE was defined as unstable angina, myocardial infarction, coronary revascularisation, or cardiac death. Data on non-fatal cardiac events were obtained through linkage of the ATTOM dataset with routinely collected hospital data (60). Death data were obtained from the UK Renal Registry and NHS Blood and Transplant. Patients were followed up until 31st December 2017, with censoring for non-cardiac deaths.

**FIGURE 3:**
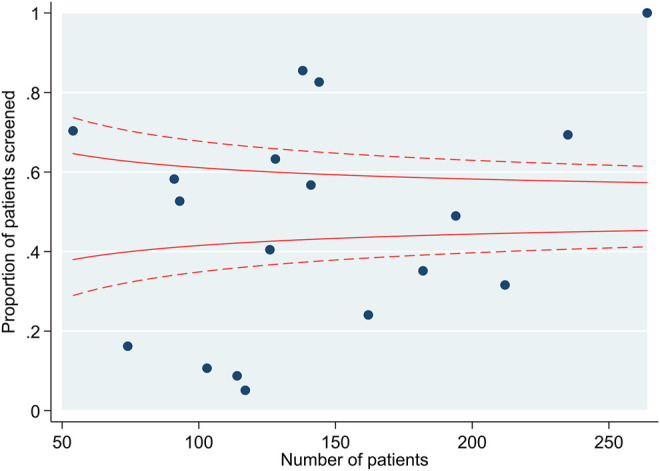
Funnel plot demonstrating the number of individuals screened by transplant centre.

Over the study period, 2572 individuals received a transplant. The mean age was 50 years (SD 13) and 61% were male. Ethnicity was White in 76%, Black in 14% and Asian in 9%. There was a history of diabetes in 13% and ischaemic heart disease in 7%. Overall, 51% underwent screening for asymptomatic coronary artery disease with a stress test (exercise tolerance test, stress echocardiogram, myocardial perfusion scan), CT coronary angiogram or invasive coronary angiogram before transplant listing. The proportion of individuals screened across the 18 transplant centres in England ranged from 5%–100% (Figure 3).

Median follow up was 5.0 years (IQR 3.8–5.5), over which time 211 individuals experienced MACE. Median time to MACE was 2.3 years (IQR 1.0–3.7; range 1 day–6.6 years). Over follow up, 227 patients died (8.9%); 40 had a cardiac death that was counted as MACE.

To examine whether screening has a causal effect on MACE at 90 days, 1 year or 5 years post-transplant, Cox regression models were performed using propensity score matching, inverse probability weighting, and instrumental variable analysis techniques.

### Competing Risks and “Direct” and “Total” Treatment Effects

Non-cardiac death is a competing risk for post-transplant MACE, as patients dying of non-cardiac causes cannot subsequently develop MACE. The analyses presented in the following section determine the “direct” effect of screening on MACE as patients are censored at non-cardiac death, as opposed to the “total” effect of screening on MACE which would include causal pathways involving non-cardiac death (61).

Interpreting direct treatment effects is challenging as they assume an unrealistic situation where competing events do not occur. Further, direct treatment effects have additional causal assumptions such as no unaccounted confounding of the relationship between the competing event (non-cardiac death) and outcome of interest (MACE). If there is likely to be a confounding relationship between the censoring event and the outcome of interest, techniques such as inverse probability of censoring weighting may be required to derive valid estimates of the direct treatment effect—such analyses require sufficient data availability for the probability of censoring (i.e., non cardiac death) to be modelled accurately over time (61).

As the purpose of this paper is to demonstrate the application of different causal inference techniques, for pragmatic reasons the following analyses represent the direct effect of screening on MACE. Information on competing risk analyses, which can navigate this issue by generating total treatment effects, are found at the following references (62, 63, 64).

### Propensity Score Matching and Inverse Probability Weighting

To generate the PS, variables deemed to potentially relate to screening and MACE were determined and included in a logistic regression model. These comprised: age, sex, ethnicity, socioeconomic status, smoking status and history of ischaemic heart disease, diabetes, cerebrovascular disease, and peripheral vascular disease. Transplant centre was not included as it should not independently associate with MACE, would prevent us capitalising on variation in practice to create groups screened and unscreened patients, and could result in violation of the positivity assumption (Supplementary Table S1).

**FIGURE 4:**
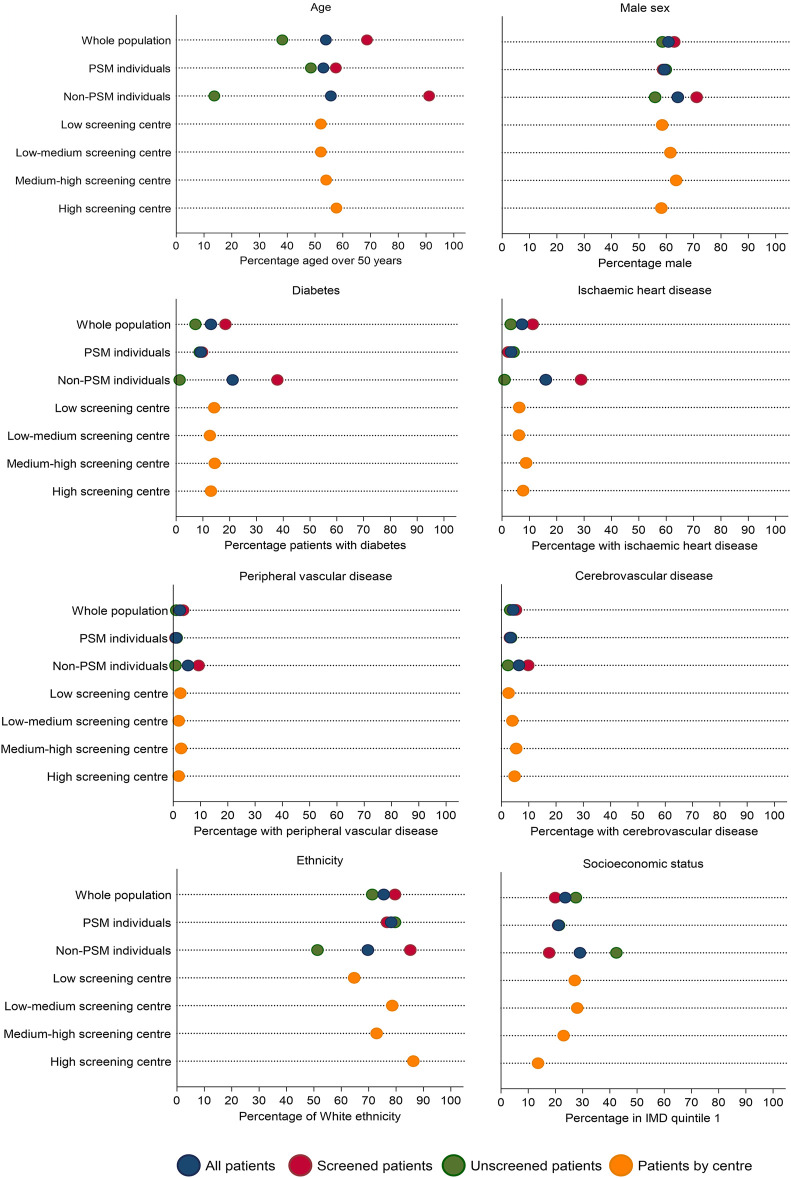
Characteristics of screened and unscreened groups across the whole population and in propensity score matched and unmatched groups, followed by characteristics by centre screening use: low volume of screening (<25% of transplant patients screened; *n* = 570), low-medium volume of screening (25%–49% screened; *n* = 714), medium-high (50%–74% screened; *n* = 742) or high volume of screening (>74% screened; *n* = 546). Note that although there is variation in patient characteristics by those screened or unscreened, this variation reduces when patients are stratified by centre screening volume, suggesting centre could be a strong instrument.

As the proportion of screened and non-screened individuals was roughly equal, PS matching was performed on a 1:1 basis without replacement using a caliper of 0.2 times the standard deviation of the logit of the propensity score. Matching was possible in 1760 individuals. The distribution of the PS before and after matching is shown in Supplementary Figure S2. The standardised mean difference after matching showed appropriate covariate balance between groups (Supplementary Table S2). The characteristics of screened and unscreened patients in PS matched and unmatched groups are shown in Figure 4. The 812 unmatched individuals were more likely to be male, of Asian ethnicity, and have a history of cardiovascular disease (Supplementary Table S3). In the PS matched population, screening did not reduce MACE at 90 days (conditional HR 0.80, 95% CI 0.31–2.05), 1 year (conditional HR 1.12, 95% CI 0.51–2.47) or 5 years (conditional HR 1.31, 95% CI 0.86–1.99) (Table 3). These results reflect the ATT: the causal effect of screening in screened patients eligible to receive either treatment (and thus “matched”), representing transplant recipients at low-medium cardiac risk.

**TABLE 3:** Association between screening and post-transplant MACE at 90 days, 1 year and 5 years using propensity score matching, weighting and instrumental variable techniques.

Association between screening and MACE at 90 days post-transplant 14 events in PS matched group, 23 events in whole population				
Method and treatment effect	HR	95% CI	*p*-value	Hazard ratio with 95% confidence interval
PS match marginal	0.75	0.33–1.72	0.50	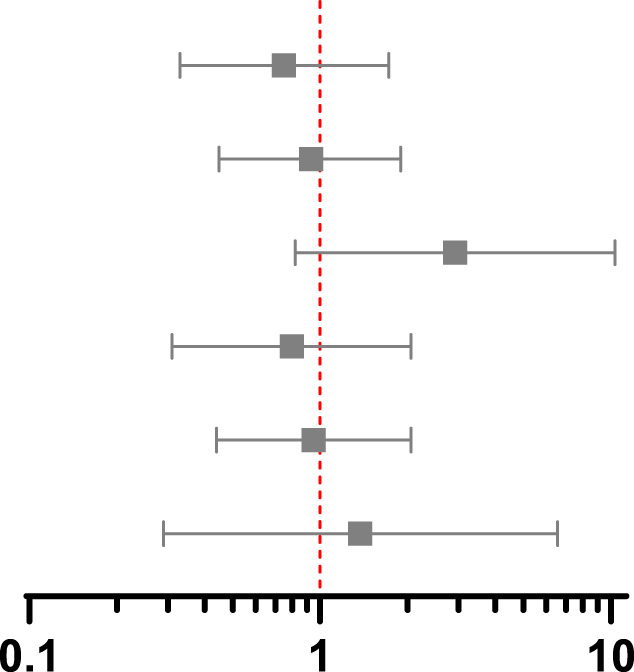
IPW marginal	0.93	0.45–1.89	0.83	
IV marginal	2.91	0.82–10.33	0.10	
PS match conditional	0.80	0.31–2.05	0.64	
IPW conditional	0.95	0.44–2.05	0.90	
IV conditional	1.37	0.29–6.55	0.69	
**Association between screening and MACE at 1 year post-transplant 32 events in PS matched group, 52 events in whole population**				
PS match marginal	1.14	0.56–2.31	0.72	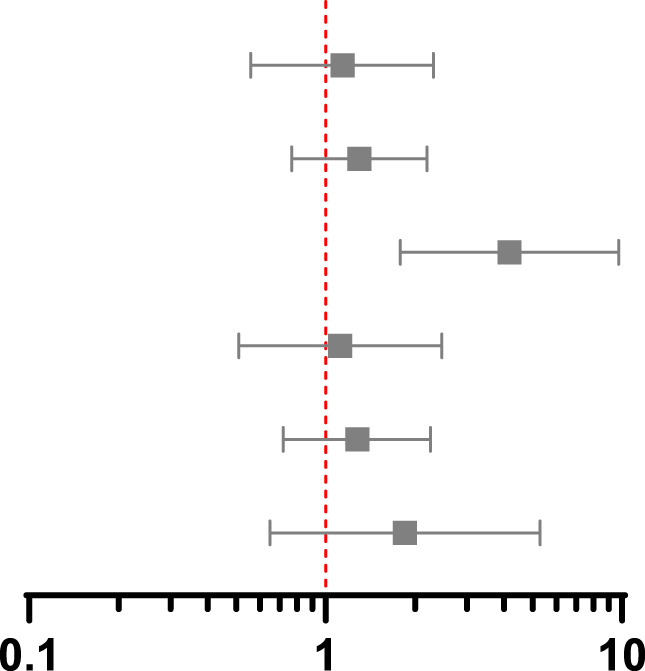
IPW marginal	1.30	0.77–2.20	0.33	
IV marginal	4.18	1.79–9.76	0.001	
PS match conditional	1.12	0.51–2.47	0.77	
IPW conditional	1.28	0.72–2.26	0.40	
IV conditional	1.85	0.65–5.29	0.25	
**Association between screening and MACE at 5 years post-transplant 117 events in PS matched group, 199 events in whole population**				
PS match marginal	1.31	0.85–2.03	0.22	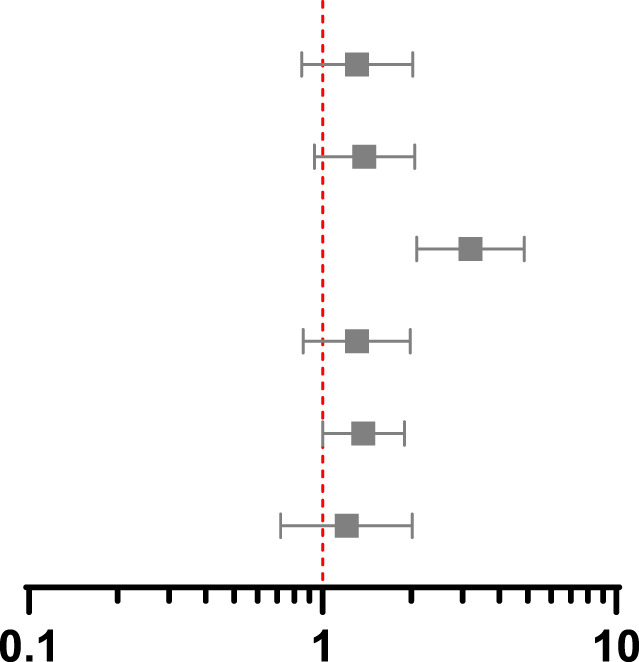
IPW marginal	1.39	0.94–2.06	0.10	
IV marginal	3.19	2.09–4.87	<0.001	
PS match conditional	1.31	0.86–1.99	0.20	
IPW conditional	1.38	1.00–1.90	0.05	
IV conditional	1.21	0.72–2.02	0.48	

CI, confidence interval; HR hazard ratio; IV, instrumental variable; PS, propensity score; IPW, inverse probability weighting. Multivariable includes variables used to estimate the propensity score in the outcome regression model.

For IPW, inverse probability of treatment weights were calculated. Weights were stabilised by multiplying them by the proportion of individuals who underwent screening in the exposed group, and proportion of individuals who did not undergo screening in the unexposed group (32). The mean of the stabilised weights was 1.00 (SD 0.47, range 0.53–8.45). Characteristics of the 57 patients with stabilised weights greater than or equal to 2 are in Supplementary Tables S4, S5. These patients were more frequently unscreened. Higher-weighted unscreened patients were older and more likely to have cardiovascular disease.

In total 2502 individuals were examined in the IPW analysis; 70 individuals were excluded due to missing data in variables used to generate the PS. Cox regression models were performed incorporating the IPW (Table 3). There was no evidence screening reduced MACE at 90 days (conditional HR 0.95, 95% CI 0.44–2.05) or 1 year (conditional HR 1.28, 95% CI 0.72–2.26). There was weak evidence that patients undergoing screening were at higher risk of MACE at 5 years (conditional HR 1.38, 95% CI 1.00–1.90), but this analysis did not meet the Cox proportionality assumption with a greater rise in MACE in screened patients over time. These results reflect the ATE: the causal effect of screening on the transplanted population. They do not provide information on the effect of screening on the total population who begin transplant workup.

It is important to note that these results represent a complete case analysis, as the 70 individuals with missing data were excluded. Complete case analyses assume data are missing completely at random, though other missing data mechanisms and their potential implications need to be considered (65).

### Instrumental Variable Analysis

Transplant centre is determined by geographical location so is largely randomly allocated. We determined centre had the potential to be an IV as it (at least partly) met the following assumptions (Figure 2B):

(1) Relevance assumption: the likelihood of undergoing screening is associated with transplant centre (Figure 4), even after adjustment for patient-level characteristics (18). On an individual patient level, screening is associated with older age, male sex, and a history of vascular disease (Supplementary Table S6) but when examining patients based on whether they are registered at a centre with a low, medium, or high screening use, differences in these variables is reduced (Table 4).
(2) Exclusion restriction: this assumption cannot be guaranteed as there could be non-screening differences in centre-level practice that influence outcome, e.g., use of medical therapy, but this would not be expected given there is national guidance on cardiovascular risk management (66), and transplant outcomes are similar between centres (67).
(3) Independence assumption: this assumption cannot be proven, as acknowledged in IV literature. Whilst it may be assumed that if measured confounders are balanced across IV groups, unmeasured confounders will be too, this is purely speculative.
(4) Homogeneity or monotonicity. Screening may not have a uniform effect on individuals, for example it could benefit those with high cardiovascular risk but not low risk patients, thus violating homogeneity. Monotonicity (no patients receiving the opposite treatment to what would be expected at any level of the instrument) may be more likely to hold as patients receive screening based on defined protocols at their transplant centre. This assumption however cannot be proven and defining the four compliance types (Supplementary Figure S1) is complex.

**TABLE 4:** Patient characteristics based on the prevalence of screening pre-transplant by centre. The Kruskall-Wallis test was used to examine continuous variables and the Chi square test for categorical variables.

Percentage of individuals screened by centre					
	<25% 4 centres *n* = 570	25%–49% 5 centres *n* = 714	50–74% 6 centres *n* = 742	≥75% 3 centres *n* = 546	*p* value
Median age (years)	50 (40–60)	50 (41–59)	52 (40–60)	52 (42–62)	0.22
Male sex (%)	58.8	61.5	63.6	58.2	0.17
White ethnicity (%)	64.7	78.6	72.9	86.3	<0.001
IMD quintile 1 (%)	27.1	28.0	23.0	13.6	<0.001
Diabetic nephropathy (%)	23.2	22.0	23.9	23.8	0.29
Diabetes (%)	14.2	12.5	14.4	10.2	0.12
Ischaemic heart disease (%)	6.3	6.2	8,8	7.7	0.20
Peripheral vascular disease (%)	2.6	2.0	2.9	2.0	0.56
Cerebrovascular disease (%)	2.6	4.0	5.4	4.8	0.09
Pre-emptive transplant (%)	20.9	20.9	24.1	20.7	0.34

BOX 1 **Selected transplant studies using propensity score and instrumental variable techniques.**

Propensity score techniques

• Comparison of outcomes in recipients receiving a living versus standard criteria deceased donor kidney transplant (74).

• Comparison of outcomes in donation after brainstem death and donation after cardiac death donors in liver transplantation (75).

• Association between immunosuppression regime (triple or quadruple therapy) in heart transplant recipients and death and rejection episodes (76).

Instrumental variable techniques

• Association between dialysis duration and patient outcome following kidney transplantation, using blood group as an instrumental variable (77).

• Examining whether delayed graft function is associated with long term outcomes after kidney transplantation using cold ischaemic time as an instrumental variable (78).

• Comparison of deceased and living organ donation rates in countries with an opt-in and opt-out policies using legal system and non-health based philanthropy as instrumental variables (79).

In the first stage, a linear regression containing potential confounders of the treatment-outcome relationship (deemed to be those used to create the PS) and transplant centre was used to predict the likelihood of an individual undergoing screening. Linear regression was selected for this analysis as opposed to logistic regression as described in IV literature (43), which also prevented individuals from centres who screened all recipients (*n* = 264) being dropped given instrument was a “perfect” predictor of outcome. Whilst using centre as an instrument addresses unmeasured patient-level confounding (i.e., unmeasured confounding between X and Y via U in Figure 2), centre-level confounding remains possible due to other institutional differences in practice (i.e., confounding between Z and Y in Figure 2 that may be distinct from U and/or C). (68) We considered including centre-specific variables which could influence outcome e.g. proportion of living donor or pre-emptive transplants, but these were not included in the final model due to collinearity with centre.

The first stage generated a predicted value, representing the likelihood of each individual being screened. The F statistic was 70 and the partial R-squared value was 0.33, indicating centre was a strong IV.

In the second stage, univariable and multivariable Cox regression models were performed using the predicted value from the first stage (predictor substitution method). This step can be considered as including the proportion of patients screened by centre as a patient characteristic, rather than whether each individual was screened. The multivariable model included the same confounders used to create the PS as these were deemed to potentially confound both the instrument-outcome and treatment-outcome relationship, and therefore including these confounders makes the independence assumption more likely to hold. Screening did not reduce MACE in the conditional model at 90 days (conditional HR 1.37, 95% CI 0.29–6.55), 1 year (conditional HR 1.85, 95% CI 0.65–5.29) or 5 years (conditional HR 1.21, 95% CI 0.72–2.02). These results reflect the LATE: the causal effect of screening on the ‘complier’ patients in the population.

### Interpretation of Results

Results from PS matched, IPW and IV analyses are shown in Table 3. In the conditional models, screening did not reduce MACE in any analysis, which each had overlapping confidence intervals, but there was variation in estimates between methods. The hazard ratios using PS methods rose over time, crossing 1 between 90 days and 1 year, whilst in the IV analysis the hazard ratio was above 1 throughout. These differences can help result interpretation by considering which patients are included in each analysis.

In the PS matched analysis, the results are only applicable to 1760 transplant recipients with low-medium baseline risk of MACE, not the overall population. The 812 individuals excluded from the analysis were more likely to be male, of Asian ethnicity, have a history of cardiovascular disease and be of a lower socioeconomic status and thus have the greatest baseline cardiovascular risk. Whilst these results suggest no benefit to screening, this cannot be directly applied to these highest risk patients.

The IPW analysis includes all patients and represents the whole transplanted population. Similar findings were observed to the PS matched analysis at 90 days and 1 year. At 5 years, there was weak evidence that individuals who had undergone screening were more likely to experience MACE in the conditional model but it should be noted that this analysis did not meet the Cox proportionality assumption.

In the IV analysis, screening did not reduce MACE on conditional analyses with a hazard ratio above 1 throughout, suggesting “complier” screened individuals had a higher risk of MACE than complier non-screened individuals, although confidence intervals were extremely wide. Given these results represent the LATE, it is not known whether the effect of screening on non-complier patients differs. Whilst the IV technique minimises unmeasured confounding, these results raise the possibility that unmeasured patient level characteristics associate with centre and outcome (i.e., clinicians screen their patients as they see their population as being inherently higher risk), or there are unmeasured differences in centre level practice, e.g., use of medical therapy that could bias results. Alternatively, it is possible that the PS matched and IPW analyses are prone to bias due to unmeasured confounding, and the IV analysis provides a result that is closer to the truth. Some studies suggest IV techniques provide less biased results than PS analyses, (69) but the challenges in identifying an appropriate instrument must be considered and results interpreted with caution until further studies examining both techniques are available (70).

The marginal hazard ratios presented in Table 3 reflect the effect of screening on the study population as opposed to an individual patient. In the PS matched and IPW analyses, screening did not reduce MACE. The results of the IV analysis differed, with screened individuals having a greater risk of MACE at 1 year (HR 4.18, 95% CI 1.79–9.76) and 5 years post-transplant (HR 3.19, 95% CI 2.09–4.87). This may reflect deviation from the independence assumption of no confounders to the instrument and outcome, the impact of which is lessened by adjusting for confounders in the conditional model.

### Limitations

Whilst the causal inference techniques applied to our worked example reduce confounding by indication, other forms of bias remain (Table 2). The worked example only examines patients who received a transplant. Data were not available for those who were screened and not listed due to an abnormal screening test, or listed but not transplanted due to MACE that occurred on the waitlist. Screening results are just one factor in a complex assessment of patients for transplantation, with the proportion of patients excluded due to cardiac screening abnormalities estimated at 1%–4% (71, 72, 73). In a target trial examining whether cardiac screening improves post-transplant outcomes these data would ideally be known, and neither PS or IV techniques specifically address this issue. Results therefore cannot be applied to the population who begin transplant workup nor determine the impact of screening on outcomes outwith post-transplant MACE.

## Summary

Propensity score and instrumental variable techniques reduce confounding in observational studies and are suited to areas where treatment decisions vary with clinician or facility preference. Whilst RCTs minimise confounding through the random allocation of treatment, results may not be generalisable if the individuals recruited to a trial are not representative of the population of interest, e.g., if individuals with less severe disease who are “lower risk” or with more severe disease who have “most to gain” are preferentially recruited. Population observational data allows all patients within clinical practice to be examined, but treatment effects from causal inference techniques still may not be applicable to the whole population due to limited overlap in confounder distributions between patient groups. Techniques deal with this issue in different ways. For example, in PS matching patients are excluded from analyses if a “suitable” match cannot be found. In IPW analyses, the presence of large weights can highlight instances where regression adjustment would result in the model being extrapolated to groups with little or no overlap in confounder distribution. Whilst large weights can make the ATE estimate unstable and results in wide confidence intervals, IPW techniques provide an “honest” reflection of the uncertainty in the estimate which might be underestimated in regression adjustment. Causal effects from each technique therefore permit inferences on different populations, which is important when interpreting study results.

Our case study demonstrates how causal inference techniques can estimate comparative effectiveness of interventions using observational data, but don’t eliminate all forms of bias and may still not allow firm conclusions to be drawn. Differences in results may reflect the different populations the estimates are applicable to, the presence of unmeasured confounding, or imperfections in the instrument. It is difficult to know which analysis provides the closest result to the “true” estimate, and results should be interpreted in the context of the limitations of each method.

Despite these challenges, the unique issues in performing RCTs in transplantation, combined with the increase in size and granularity of routine healthcare datasets are likely to result in wider use of propensity score and instrumental variable techniques. Examples of transplantation studies using these techniques are shown in Box 1. There is potential to explore areas such as the optimal timing of pre-emptive transplantation, identifying which patients may benefit from transplantation, and how outcomes differ based on donor type. By identifying areas where there is variation in practice and clinical equipoise, these analyses can provide preliminary data to guide clinical trials. We welcome the possibility of this in the field of cardiac screening prior to kidney transplant listing.

## Abbreviations

ATE, average treatment effect; ATT, average treatment effect on the treated; ATTOM, Access to Transplant and Transplant Outcome Measures Study; CI, confidence interval; HR, Hazard ratio; IMD, index of multiple deprivation; IV, instrumental variable; IPW, inverse probability weighting (using propensity scores); IQR, interquartile range; LATE, local average treatment effect; MACE, major adverse cardiac event; PS, propensity score; RCT, randomised controlled trial; SD, standard deviation.

## Glossary

Propensity score: a value ranging between 0 and 1 that summaries the likelihood of an individual receiving a treatment based on their measured covariates
Propensity score matching: process through which individuals in treated and untreated groups are matched to each other based on their propensity score. This can be done on a 1:1 (1 patient in the untreated group matched to 1 treated individual) or many-to-one (many patients in the untreated group matched to 1 treated individual) basis
Matching without replacement: once an individual from the untreated group has been matched, they cannot be used as a comparator for any further treated individuals
Matching with replacement: an individual in the untreated group can be used as a match for more than 1 treated individual. Useful if the number of untreated individuals is small.
Nearest neighbour matching: matching process which pairs treated and untreated individuals based on them having the closest propensity scores, irrespective of whether the untreated individual is a better match for another treated individual.
Optimal matching: matching process which aims to minimise the difference in propensity scores between pairs across the whole population. May be preferred over nearest neighbour matching if the proportion of untreated individuals in the population is small.
Inverse probability weighting: technique which weights individuals based on their propensity score to create a pseudo-population with balanced measured covariates in treated and untreated groups
Instrumental variable: a variable that is causally associated with the exposure, only affects outcome through its association with that exposure, and has no confounders with the outcome. Allows individuals to be examined based on the instrument to minimise the risk of unmeasured confounding.
