# Supplementary material for: Propensity Score and Instrumental Variable Techniques in Observational Transplantation Studies: An Overview and Worked Example Relating to Pre-Transplant Cardiac Screening
Source: Transpl Int. 2022 Jun 27;35:10105. doi: 10.3389/ti.2022.10105 (PMC9271574; doi:10.3389/ti.2022.10105)
Supplement: Supplementary file 1 [file DataSheet1.docx]

**Supplementary Material**

**Supplementary Tables**

| Assumption | Definition |
| --- | --- |
| Exchangeability/ignorability ^S1^ ^S2^ | The treatment which an individual receives is unrelated to their potential outcome i.e. the patients in treated and untreated groups have the same distribution of outcome predictors, and would have the same distribution of outcomes if they all received the treatment of interest. This assumption is violated if individuals who are likely to have a good outcome regardless of treatment are more likely to receive treatment. |
| Positivity ^S3^ | All subgroups of individuals in a covariate stratum have a non-zero chance of receiving either treatment option i.e. within each covariate subgroup, it must be possible for patients to receive either of the treatment options. |
| Consistency ^S4^ ^S5^ | This assumes that the exposure is well defined and has a stable/consistent impact on outcome. This could be violated if, for example, the outcome varies depending on how treatment is delivered. |

Table S1. Description of assumptions in propensity score matching and inverse probability weighting techniques.

|  |  | Unmatched characteristics  Exposure to CAD Screening | | | Propensity-score matched  Exposure to CAD Screening | | |
| --- | --- | --- | --- | --- | --- | --- | --- |
|  |  | **No** | **Yes** | **SMD** | **No** | **Yes** | **SMD** |
| Age (years)  Median [IQR] |  | 46  [36-55] | 56  [47-63] | 0.7 | 49  [42-58] | 52  [43-57] | 0.05 |
| Male |  | 59% | 63% | 0.09 | 60% | 59% | 0.02 |
| Ethnicity | White  Asian  Black  Mixed | 71%  17%  10%  2% | 80%  11%  8%  2% | 0.20 | 80%  11%  8%  1% | 77%  12%  10%  2% | 0.13 |
| IMD | 1  2  3  4  5 | 27%  20%  19%  17%  17% | 20%  21%  19%  22%  18% | 0.19 | 21%  19%  19%  21%  20% | 21%  23%  22%  20%  15% | 0.17 |
| PRD | GN  Other  PKD  Uncertain  PN  Diabetes  Hypertension  Renovascular | 26%  23%  16%  12%  12%  4%  6%  1% | 22%  20%  18%  2%  9%  12%  6%  11% | 0.33 | 26%  19%  19%  11%  11%  6%  7%  1% | 24%  21%  19%  12%  10%  6%  7%  1% | 0.11 |
| Diabetes |  | 7% | 18% | 0.34 | 10% | 9% | 0.03 |
| IHD |  | 3% | 11% | 0.31 | 4% | 2% | 0.11 |
| PVD |  | 1% | 4% | 0.17 | 1% | 1% | 0.05 |
| CeVD |  | 3% | 5% | 0.10 | 4% | 3% | 0.03 |
| Ever smoker |  | 29% | 36% | 0.14 | 34% | 29% | 0.12 |
| RRT Modality | HD  PD  Transplant  Pre-emptive | 57%  21%  1%  21% | 60%  18%  1%  21% | 0.11 | 57%  22%  1%  20% | 58%  20%  1%  21% | 0.10 |

Table S2. Standardised mean differences before and after propensity score matching. Abbreviations: IMD, index of multiple deprivation; PRD, primary renal diagnosis; GN, glomerulonephritis; PKD, polycystic kidney disease; PN, pyelonephritis; IHD, ischaemic heart disease; PVD, peripheral vascular disease; CeVD, cerebrovascular disease; RRT, renal replacement therapy; HD, haemodialysis; PD, peritoneal dialysis.

|  | Propensity matched  N=1760 | Not propensity matched  N=812 | P |
| --- | --- | --- | --- |
| Age (years) (n=2572) | 50 [43 – 58] | 54 [34 – 64] | 0.09 |
| Male Sex (n=2572) | 1043 (59) | 521 (64) | 0.02 |
| Ethnicity (n=2563)  White  Asian  Black  Mixed | 1376 (78)  203 (12)  161 (9)  20 (1) | 566 (70)  154 (19)  62 (8)  21 (3) | <0.001 |
| PRD (n=2555)  GN  Other  PKD  Uncertain  PN  Diabetes  Hypertension  Renovascular | 437 (25)  349 (20)  335 (19)  202 (11)  189 (11)  103 (6)  121 (7)  15 (1) | 174 (22)  193 (24)  90 (11)  105 (13)  79 (10)  111 (14)  39 (5)  13 (1) | <0.001 |
| History of Diabetes (n=2572) | 162 (9) | 171 (21) | <0.001 |
| History of IHD (n=2572) | 57 (3) | 130 (16) | <0.001 |
| History of PVD (n=2572) | 18 (1) | 44 (5) | <0.001 |
| History of CeVD (n=2572) | 58 (3) | 52 (6) | <0.001 |
| Ever smoker (n=2507) | 563 (32) | 261 (35) | 0.15 |
| RRT modality (n=2556)  HD  PD  Transplant  Pre-emptive | 1010 (58)  364 (21)  15 (1)  361 (20) | 482 (60)  140 (17)  3 (1)  181 (22) | 0.09 |
| IMD (n=2572)  1 – Most deprived  2  3  4  5 – Least deprived | 372 (21)  367 (21)  360 (20)  356 (20)  305 (17) | 235 (29)  151 (19)  130 (16)  149 (18)  147 (18) | <0.001 |
| Centre (anonymised) (n=2572)  1  2  3  4  5  6  7  8  9  10  11  12  13  14  15  16  17  18 | 129 (7)  81 (5)  211 (12)  119 (7)  49 (3)  85 (5)  59 (3)  162 (9)  80 (5)  98 (6)  76 (4)  161 (9)  97 (6)  53 (3)  111 (6)  35 (2)  92 (50  62 (4) | 53 (7)  60 (7)  53 (7)  43 (5)  25 (3)  41 (5)  44 (5)  73 (9)  34 (4)  46 (6)  52 (6)  51 (6)  41(5)  40 (5)  83 (10  19 (2)  25 (3)  29 (4) | <0.001 |
| Live donor (n=2572) | 520 (30) | 251 (31) | 0.48 |
| Screening (n=2572) | 880 (50) | 440 (54) | 0.05 |

Table S3. Patient and transplant characteristics in propensity score matched and unmatched individuals. Data are expressed as number (%) or median [interquartile range]. Abbreviations: IMD, index of multiple deprivation; PRD, primary renal diagnosis; GN, glomerulonephritis; PKD, polycystic kidney disease; PN, pyelonephritis; IHD, ischaemic heart disease; PVD, peripheral vascular disease; CeVD, cerebrovascular disease; RRT, renal replacement therapy; HD, haemodialysis; PD, peritoneal dialysis.

|  | Screened patients stabilised weight <2  N= 1272 | Screened patients stabilised weight > 2  N= 15 | P value |
| --- | --- | --- | --- |
| Age (years) (n=1287) | 56 [47-63] | 33 [26-36] | <0.001 |
| Male Sex (n=1287) | 798 (63) | 6 (40) | 0.07 |
| Ethnicity  White  Asian  Black  Mixed | 1027 (81)  134 (11)  95 (7)  16 (1) | 2 (13)  7 (47)  5 (33)  1 97) | <0.001 |
| History of Diabetes (n=1287) | 240 (19) | 0 (0) | 0.06 |
| History of IHD (n=1287) | 145 (11) | 0 (0) | 0.17 |
| History of PVD (n=1287) | 48 (4) | 0 (0) | 0.44 |
| History of CeVD (n=1287) | 67 (5) | (0) | 0.36 |
| Ever smoker (n=1287) | 464 (36) | 1 (7) | 0.02 |
| IMD (n=1287)  1 – Most deprived  2  3  4  5 – Least deprived | 244 (19)  264 (21)  247 (19)  280 (22)  237 (19) | 8 (53)  2 (13)  2 (13)  2 (13)  1 (7) | 0.03 |

Table S4. Characteristics of screened patients (n=1287) with stabilised weights under 2 and 2 or greater. Data are expressed as number (%) or median [interquartile range]. Abbreviations: IMD, index of multiple deprivation; IHD, ischaemic heart disease; PVD, peripheral vascular disease; CeVD, cerebrovascular disease.

|  | Unscreened patients stabilised weight <2  N=1173 | Unscreened patients stabilised weight > 2  N=42 | P value |
| --- | --- | --- | --- |
| Age (years) (n=1215) | 45 [35-54] | 65 [63-68] | <0.001 |
| Male Sex (n=1215) | 681 (58) | 30 (71) | 0.08 |
| Ethnicity (n=1215)  White  Asian  Black  Mixed | 834 (71)  199 (17)  117 (10)  23 (2) | 31 (74)  8 (19)  3 (7)  0 (0) | 0.73 |
| History of Diabetes (n=1215) | 58 (5) | 30 (71) | <0.001 |
| History of IHD (n=1215) | 21 (2) | 17 (40) | <0.001 |
| History of PVD (n=1215) | 7 (1) | 6 (14) | <0.001 |
| History of CeVD (n=1215) | 37 (3) | 3 (7) | 0.16 |
| Ever smoker (n=1215) | 342 (29) | 15 (36) | 0.36 |
| IMD (n=1215)  1 – Most deprived  2  3  4  5 – Least deprived | 327 (28)  225 (19)  225 (19)  204 (17)  192 (16) | 7 (17)  12 (29)  4 (9)  8 (19)  11 (26) | 0.09 |

Table S5. Characteristics of unscreened patients (n=1215) with stabilised weights under 2 and 2 or greater. Data are expressed as number (%) or median [interquartile range]. Abbreviations: IMD, index of multiple deprivation; IHD, ischaemic heart disease; PVD, peripheral vascular disease; CeVD, cerebrovascular disease.

|  | Unadjusted OR  (95% CI) | P | Adjusted OR  (95% CI) | P |
| --- | --- | --- | --- | --- |
| Age (years) | 1.05 (1.05 – 1.06) | <0.001 | 1.08 (1.07 – 1.09) | <0.001 |
| Male sex (Ref: Female) | 1.20 (1.02 – 1.40) | 0.03 | 1.22 (0.97 – 1.54) | 0.10 |
| Ethnicity (Ref: White)  Asian  Black  Mixed | 0.59 (0.47 – 0.75)  0.70 (0.53 – 0.93)  0.66 (0.36 – 1.24) | <0.001  0.01  0.20 | 0.75 (0.53 – 1.08)  0.61 (0.40 – 0.94)  0.60 (0.25 – 1.42) | 0.13  0.03  0.25 |
| PRD (Ref: PN)  GN  Other  Uncertain  PKD  Diabetes  Hypertension  Renovascular | 1.12 (0.84 – 1.49)  1.16 (0.87 – 1.56)  1.23 (0.89 – 1.71)  1.49 (1.09 – 2.02)  3.67 (2.49 – 5.43)  1.41 (0.95 – 2.08)  4.66 (0.89 – 1.71) | 0.45  0.31  0.22  0.01  <0.001  0.09  0.22 | 0.75 (0.49 – 1.14)  1.13 (0.74 – 1.74)  0.95 (0.59 – 1.53)  0.78 (0.50 – 1.20)  1.69 (0.81 – 3.55)  1.13 (0.65 – 1.97)  1.03 (0.26 – 4.19) | 0.18  0.57  0.83  0.25  0.16  0.66  0.96 |
| Diabetes (Ref: Absent) | 2.91 (2.26-3.76) | <0.001 | 3.11 (1.84 – 5.25) | <0.001 |
| IHD (Ref: Absent) | 3.80 (2.65 – 5.44) | <0.001 | 2.93 (1.76 – 4.86) | <0.001 |
| PVD (Ref: Absent) | 3.34 (1.83 – 6.08) | <0.001 | 1.70 (0.74 – 3.91) | 0.21 |
| CeVD (Ref: Absent) | 1.70 (1.14 – 2.52) | 0.007 | 0.62 (0.35 – 1.08) | 0.09 |
| Ever Smoker (Ref: Never) | 1.36 (1.15 – 1.60) | <0.001 | 1.12 (0.88 – 1.43) | 0.37 |
| RRT Modality (Ref: HD)  PD  Transplant  Pre-emptive | 0.83 (0.67 – 1.01)  0.35 (0.12 – 0.98)  0.98 (0.80 – 1.19) | 0.06  0.05  0.82 | 0.84 (0.63 – 1.13)  0.29 (0.08 – 1.11)  1.07 (0.80 – 1.43) | 0.26  0.11  0.69 |
| IMD (Ref: 1)  2  3  4  5 | 1.43 (1.13 – 1.82)  1.43 (1.13 – 1.82)  1.72 (1.36 – 2.19)  1.52 (1.19 – 1.94) | 0.003  0.003  <0.001  0.001 | 1.17 (0.83 – 1.64)  0.92 (0.65 – 1.32)  1.19 (0.84 – 1.70)  0.90 (0.62 – 1.31) | 0.38  0.67  0.33  0.58 |
| Centre (Ref: Bristol)  1  2  3  4  5  6  7  8  9  10  11  12  13  14  15  16 | 0.41 (0.26-0.65)  0.24 (0.15 – 0.39)  0.15 (0.07 – 0.30)  0.52 (0.32 – 0.84)  1.73 (1.12 – 2.66)  0.09 (0.04 – 0.19)  3.63 (2.10-6.25)  0.07 (0.04 – 0.15)  1.31 (0.81 – 2.24)  0.35 (0.23 – 0.55)  4.50 (2.52 – 8.03)  0.85 (0.50 – 1.44)  0.73 (0.47 – 1.13)  1.81 (0.92 – 3.55)  0.04 (0.02 – 0.10)  1.06 (0.62 – 1.81) | <0.001  <0.001  <0.001  0.008  0.01  <0.001  <0.001  <0.001  0.28  <0.001  <0.001  0.54  0.16  0.08  <0.001  0.82 | 0.35 (0.20 – 0.6q)  0.16 (0.09 – 0.29)  0.07 (0.03 – 0.16)  0.43 (0.25 – 0.83)  2.39 (1.37 – 4.14)  0.06 (0.03 – 0.14)  4.52 (2.37 – 8.62)  0.03 (0.01 – 0.06)  1.26 (0.67 – 2.35)  0.27 (0.16 – 0.47)  5.70 (2.90 – 11.21)  0.72 (0.37 – 1.40)  0.47 (0.27 – 0.82)  1.59 (0.70 – 3.63)  0.01 (0.01-0.04)  0.93 (0.48 – 1.79) | <0.001  <0.001  <0.001  0.01  0.002  <0.001  <0.001  <0.001  0.48  <0.001  <0.001  0.38  0.008  0.27  <0.001  0.82 |

Table S6. Logistic regression of factors associated with screening. One transplant centre was removed as all patients underwent screening. Abbreviations: PRD, primary renal diagnosis; GN, glomerulonephritis; PKD, polycystic kidney disease; PN, pyelonephritis; IHD, ischaemic heart disease; PVD, peripheral vascular disease; CeVD, cerebrovascular disease; RRT, renal replacement therapy; HD, haemodialysis; PD, peritoneal dialysis.

**Supplementary Figures**

Figure S1. Figure demonstrating the four compliance types that patients can follow, as used within instrumental variable analysis terminology. Although this illustration implies the distinction between these groups is clear-cut, in reality they are poorly defined, particularly when the instrument is multi-categorical as in the worked example of this paper.


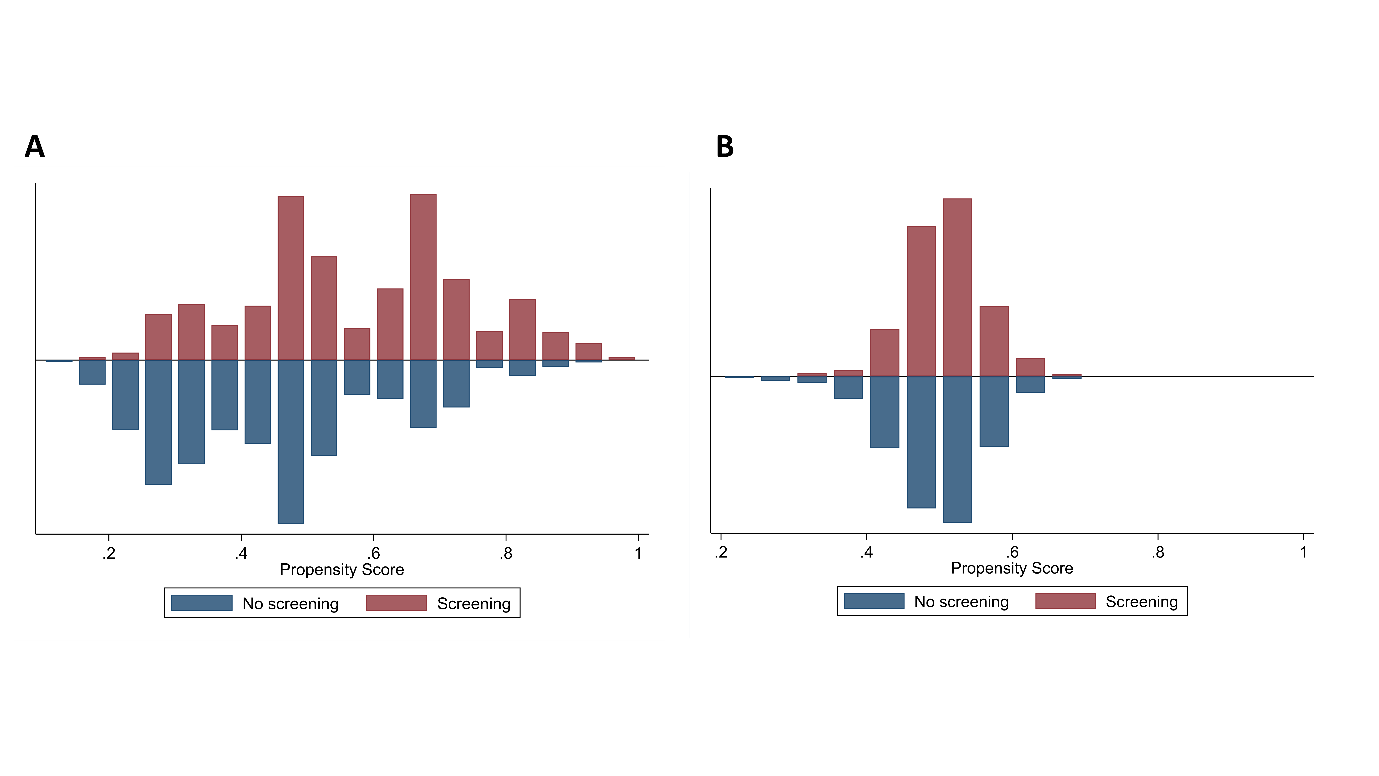


Figure S2. Balance of propensity scores before and after matching.

**Supplementary References**

S1. GREENLAND S, ROBINS JM. Identifiability, Exchangeability, and Epidemiological Confounding. *International Journal of Epidemiology*. 1986;15(3):413-419. doi:10.1093/ije/15.3.413

S2. Greenland S, Robins JM. Identifiability, exchangeability and confounding revisited. *Epidemiol Perspect Innov*. 2009;6:4-4. doi:10.1186/1742-5573-6-4

S3. Westreich D, Cole SR. Invited Commentary: Positivity in Practice. *American Journal of Epidemiology*. 2010;171(6):674-677. doi:10.1093/aje/kwp436

S4. VanderWeele TJ. Concerning the Consistency Assumption in Causal Inference. *Epidemiology*. 2009;20(6). https://journals.lww.com/epidem/Fulltext/2009/11000/Concerning_the_Consistency_Assumption_in_Causal.18.aspx

S5. Hernán MA. Does water kill? A call for less casual causal inferences. *Ann Epidemiol*. 2016;26(10):674-680. doi:10.1016/j.annepidem.2016.08.016
